# Supplementary material for: COZOID: contact zone identifier for visual analysis of protein-protein interactions
Source: BMC Bioinformatics. 2018 Apr 6;19:125. doi: 10.1186/s12859-018-2113-6 (PMC5889581; doi:10.1186/s12859-018-2113-6)
Supplement: Supplementary file 2 — Software build. Executable binary file of the software tool. (ZIP 118,784 kb) [file 12859_2018_2113_MOESM2_ESM.zip › cozoid/java/docs/beansbinding-1.2.1-doc.zip!/index-files/index-4.html]

D-Index (Beans Binding)


---


|  |  |  |
| --- | --- | --- |
| **Overview** \| Package \| Class \| Use \| **Tree** \| **Deprecated** \| **Index** \| **Help** | |  |
| **PREV LETTER**   **NEXT LETTER** | **FRAMES**    **NO FRAMES**     **All Classes** |  |


A B C D E F G H I J K L M N O P Q R S T U V W 

---


## **D**

**debugStream** - Variable in class org.jdesktop.el.impl.parser.ELParserTokenManager: **DEFAULT** - Static variable in interface org.jdesktop.el.impl.parser.ELParserConstants: **DeferredExpression()** - Method in class org.jdesktop.el.impl.parser.ELParser: **DIGIT** - Static variable in interface org.jdesktop.el.impl.parser.ELParserConstants: **disable\_tracing()** - Method in class org.jdesktop.el.impl.parser.ELParser: **DIV0** - Static variable in interface org.jdesktop.el.impl.parser.ELParserConstants: **DIV1** - Static variable in interface org.jdesktop.el.impl.parser.ELParserConstants: **divide(Number, Number)** - Method in class org.jdesktop.el.impl.lang.ELArithmetic.BigDecimalDelegate: **divide(Number, Number)** - Method in class org.jdesktop.el.impl.lang.ELArithmetic.BigIntegerDelegate: **divide(Object, Object)** - Static method in class org.jdesktop.el.impl.lang.ELArithmetic: **divide(Number, Number)** - Method in class org.jdesktop.el.impl.lang.ELArithmetic: **divide(Number, Number)** - Method in class org.jdesktop.el.impl.lang.ELArithmetic.DoubleDelegate: **divide(Number, Number)** - Method in class org.jdesktop.el.impl.lang.ELArithmetic.LongDelegate: **Done()** - Method in class org.jdesktop.el.impl.parser.SimpleCharStream: **DOT** - Static variable in interface org.jdesktop.el.impl.parser.ELParserConstants: **DotSuffix()** - Method in class org.jdesktop.el.impl.parser.ELParser: **DOUBLE** - Static variable in class org.jdesktop.el.impl.lang.ELArithmetic: **dump(String)** - Method in class org.jdesktop.el.impl.parser.SimpleNode: **DynamicExpression()** - Method in class org.jdesktop.el.impl.parser.ELParser

---


|  |  |  |
| --- | --- | --- |
| **Overview** \| Package \| Class \| Use \| **Tree** \| **Deprecated** \| **Index** \| **Help** | |  |
| **PREV LETTER**   **NEXT LETTER** | **FRAMES**    **NO FRAMES**     **All Classes** |  |


A B C D E F G H I J K L M N O P Q R S T U V W 

---
